# Botulinum toxin injection changes resting state cerebellar connectivity in cervical dystonia

**DOI:** 10.1038/s41598-021-87088-z

**Published:** 2021-04-15

**Authors:** Pavel Hok, Lenka Hvizdošová, Pavel Otruba, Michaela Kaiserová, Markéta Trnečková, Zbyněk Tüdös, Petr Hluštík, Petr Kaňovský, Martin Nevrlý

**Affiliations:** 1 Department of Neurology, University Hospital Olomouc, I. P. Pavlova 6, 77900 Olomouc, Czech Republic; 2 Department of Neurology, Faculty of Medicine and Dentistry of Palacký University Olomouc, Olomouc, Czech Republic; 3 Department of Radiology, University Hospital Olomouc, Olomouc, Czech Republic; 4 Department of Radiology, Faculty of Medicine and Dentistry of Palacký University Olomouc, Olomouc, Czech Republic; 5 Department of Computer Science, Faculty of Science of Palacký University Olomouc, Olomouc, Czech Republic

**Keywords:** Neuroscience, Neurology

## Abstract

In cervical dystonia, functional MRI (fMRI) evidence indicates changes in several resting state networks, which revert in part following the botulinum neurotoxin A (BoNT) therapy. Recently, the involvement of the cerebellum in dystonia has gained attention. The aim of our study was to compare connectivity between cerebellar subdivisions and the rest of the brain before and after BoNT treatment. Seventeen patients with cervical dystonia indicated for treatment with BoNT were enrolled (14 female, aged 50.2 ± 8.5 years, range 38–63 years). Clinical and fMRI examinations were carried out before and 4 weeks after BoNT injection. Clinical severity was evaluated using TWSTRS. Functional MRI data were acquired on a 1.5 T scanner during 8 min rest. Seed-based functional connectivity analysis was performed using data extracted from atlas-defined cerebellar areas in both datasets. Clinical scores demonstrated satisfactory BoNT effect. After treatment, connectivity decreased between the vermis lobule VIIIa and the left dorsal mesial frontal cortex. Positive correlations between the connectivity differences and the clinical improvement were detected for the right lobule VI, right crus II, vermis VIIIb and the right lobule IX. Our data provide evidence for modulation of cerebello-cortical connectivity resulting from successful treatment by botulinum neurotoxin.

## Introduction

Botulinum neurotoxin A (BoNT) injections are currently the preferred, even if symptomatic, treatment of focal dystonia^28^. Although the primary BoNT site of action is at the neuromuscular junction, the clinical effect in dystonia is assumed to be mediated by dynamic changes at multiple levels of the sensorimotor system, which was demonstrated in several neurophysiological^1,23,33^, functional MRI^38,41^ and clinical studies e.g.,^14,22,32^.

In idiopathic cervical dystonia (CD), no consistent morphological tissue abnormalities have so far been observed in structural quantitative MRI or histopathological studies^3,25^. However, functional MRI (fMRI) evidence demonstrates changes in multiple resting state networks (e.g.,^12^, which partly normalize with botulinum neurotoxin A (BoNT) therapy, suggesting primarily functional disruption of the motor control^25,34^. Nevertheless, there are only a few publications discussing resting state fMRI in CD^9,11,12,30,35,40,51^. These studies demonstrate functional connectivity changes at either cortical or subcortical levels thought to reflect defective planning, disturbed spatial cognition, and compensatory executive control of accurate movements^12^.

Recently, the role of the cerebellum in the pathophysiology of dystonia has been discussed^2,9,20,21,50,52^. Prominent cerebellar involvement has been reported both in task-related^20^ and in resting-state ^9^ fMRI studies. Interestingly, cerebellum was the only region showing consistent post-mortem histopathological changes across 6 patients with CD^45^ and one of the two brain areas specifically associated with secondary CD^9^. However, the effects of BoNT-A on resting state cerebello-cortical connectivity have not yet been investigated in sufficient detail. The only studies assessing the effect of BoNT-A on resting state connectivity either did not evaluate any cerebellar regions of interest^6^ or evaluated the cerebellum as a single region of interest (ROI)^12^. The remaining studies of resting-state connectivity in CD did not assess the effects of treatment and included patients with different times since their previous BoNT-A injection^35,40^. The lack of detailed analysis of treatment-related changes in cerebellar functional connectivity thus poses a surprisingly significant knowledge gap, especially considering the potentially important role of the cerebellum in the pathophysiology of dystonia.

Therefore, the aim of our study was to compare the whole-brain functional connectivity of cerebellar regions before and after treatment initiation. Due to expected variability in the clinical effect of the first BoNT-A injection^31^, the analysis focused on a relationship between the functional connectivity and the individual amount of clinical improvement.

## Methods

The diagnosis of CD was determined following a comprehensive neurological examination by a movement disorders specialist, based on history of typical clinical symptoms for at least 12 months and polyelectromyographic examination of neck muscles. All subjects had a recent magnetic resonance imaging (MRI) of the brain with no structural abnormality. Each patient was informed in detail about the goal and the course of investigation and signed an informed consent form. The study protocol was approved by the local ethics committee (Ethics Committee of the University Hospital and the Faculty of Medicine and Dentistry of Palacký University Olomouc, Czech Republic), in accordance with the principles and recommendations of the Declaration of Helsinki, 1975 and later revisions.

### Patients

Seventeen patients with CD indicated for treatment with BoNT were enrolled (14 female, aged 50.2 ± 8.5 years, range 38–63 years). Clinical and functional MRI examinations were carried out immediately before and 4 weeks after the first BoNT injection.

The severity of CD was evaluated using the Toronto Western Spasmodic Torticollis Rating Scale (TWSTRS)^8^ at two sessions: at Week 0 (W0—on the day of the first fMRI examination immediately before the BoNT injection) and at Week 4 (W4- on the day of the second fMRI examination four weeks after the BoNT injection). The BoNT treatment was indicated and carried out according to national and international standards and there was no investigational treatment involved. In all patients, the injected muscles were determined on the basis of a polyelectromyographic examination, provided by 4-channel Keypoint workstation, Medtronic, Minneapolis, MN, USA. The details of the electromyographic examination and BoNT injection were described in our previous work^33^. All patients were treated with onabotulinum toxin type A (Botox,Allergan, Inc, Irvine, CA, USA in concentrations of 25 IU/ml. The demographic and clinical data of the patients are presented in Table 1.

**Table 1:** Demographic and clinical data of subjects.

Sex	Age	Total BoNT-A dose (Botox)	TWSTRS at Week 0	TWSTRS at Week 4
F	39	150	15	6
F	55	200	11	5
F	38	100	12	4
F	50	200	16	6
F	47	200	16	6
M	49	150	12	6
F	61	150	13	10
F	49	125	14	12
F	60	200	21	13
F	60	200	18	13
F	44	200	13	13
F	58	200	10	5
F	63	175	13	12
M	43	100	17	11
F	40	125	14	9
F	56	175	18	10
M	41	100	10	7
Mean	50.2	161.8	14.3	8.7

List of 17 subjects with thein sex and age, total dose of BoNT used for single application and score of TWSTRS at W0 and W4. Abbreviations: F—female, M-male.

### Data acquisition

The acquisition of MRI data was performed at W0 and W4 visits using 1.5-T scanners (Siemens Aera, Avanto, and Symphony, Erlangen, Germany) with standard head coils. To avoid any possible effects due to scanner used, the schedule was either matched or counter-balanced. To provide maximum comfort and minimize head motion, the patient’s head was immobilized with cushions. During the acquisition, patients were asked to lie still with their eyes closed and not to think about anything in particular. The MRI protocol consisted of a functional T_2_^*^-weighted BOLD image acquisition using a gradient-echo echo-planar imaging (EPI) sequence with 30 axial slices parallel to the anterior commissure-posterior commissure line, 5 mm slice thickness, repetition time/echo time (TR/TE) = 2500/40 ms, flip angle 80°, field of view (FoV) = 220 mm, matrix 64 × 64, resolution 3.4 × 3.4 × 5.0 mm, 192 volumes. Furthermore, gradient-echo phase and magnitude field map images with identical geometry were acquired to allow correction of the B_0_ imaging distortions. For anatomical reference, a high-resolution three-dimensional MPRAGE scan was also acquired.

### Data Pre-processing

Initially, the fMRI data were checked for susceptibility or severe motion artifacts, but no subject had to be excluded. The statistical analysis of BOLD time-series was performed in FEAT Version 6.00, part of FSL (FMRIB’s Software Library, www.fmrib.ox.ac.uk/fsl), version 5.0.9^29^. The built-in pre-processing pipeline included: correction of B_0_ distortions, motion correction using MCFLIRT, non-brain removal, and spatial smoothing using a Gaussian kernel with 8.0 mm FWHM. In each patient, affine registration matrices between the functional images from either session and a single anatomical reference image were obtained. The anatomical reference was chosen based on a quality assessment of both available T_1_-weighted scans. Finally, a non-linear registration of the anatomical image to the MNI 152 standard space using FNIRT was calculated^24^. Next, residual motion-related artifacts were regressed out from functional time-series using ICA-AROMA noise component classification tool^48^ and high-pass temporal filtering with sigma = 60.0 s was applied. In a parallel pre-processing pipe-line, the same ICA-AROMA noise components were regressed from data with no spatial smoothing applied. This unsmoothed dataset was used to extract both the signal of interest and non-neuronal nuisance signal.

### Extraction of time-series

**Figure 1:**
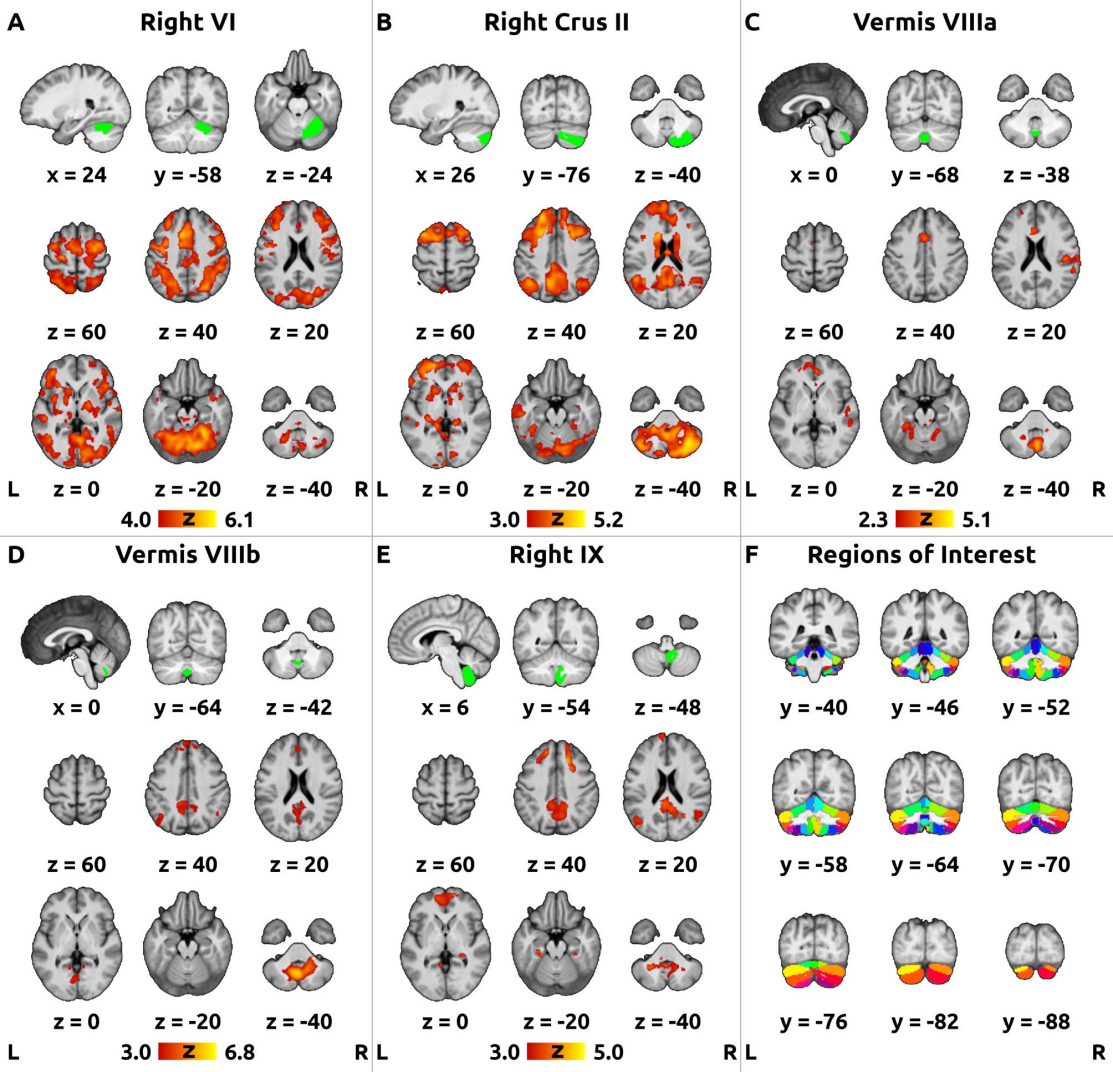
Mean functional connectivity of selected regions of interest. Figure shows mean *post-hoc* functional connectivity averaged across both sessions (baseline + follow-up) for the seeds with significant treatment-related changes and correlations in the main analysis: (**A**) Right Lobule VI, (b) Right Crus II, (**C**) Vermis VIIIa, (**D**) Vermis VIIIb, and (**E**) Right Lobule IX. The green overlays in the top row of panels A-E show the respective regions of interest (ROI) in the cerebellum. The red-yellow overlays in the bottom rows of panels A-E show the functional connectivity of each ROI across the whole brain, thresholded at different cluster-forming levels to produce visually comparable statistical maps (panel A: Z > 4.0; panels B, D,E: Z > 3.0; panel C: Z > 2.3) with the corrected cluster significance level of *p* < 0.05. An average T_1_-weighted image with the MNI152 standard space coordinates was used as a background. Right is right according to neurological convention.

The time-series for seed-based connectivity were extracted using anatomically defined ROIs based on the FSL built-in Probabilistic Cerebellar Atlas parcellation with 50% probability threshold^15^, see Fig. 1, Panel F. At this threshold, mask of label 9 (Vermis Crus I) contained 0 voxels and was therefore discarded. Next, binarized masks were transformed into each individual’s functional space using an inverted non-linear transformation field estimated in the pre-processing pipeline, resampled using trilinear interpolation and thresholded at 0.5. After the resampling, mask of label 15 (Vermis VIIb) contained 0 voxels in number of subjects and was discarded as well. The final set of ROIs consisted therefore of 26 regions. In each of the ROIs, the first eigenvariate was extracted and used as a representative de-meaned time-series.

To provide a broader context for the changes in the cerebellar networks, we performed a supplementary exploratory connectivity analysis using several regions of interest in the cerebral cortex and basal ganglia selected a priori as a reference. The specific procedures of the supplementary analysis are described in Supplementary Methods, the remaining procedures were identical to the main analysis.

Additionally, nuisance signal from six sources in the white matter and one source in the lateral ventricles was obtained as described elsewhere^27^.

### Statistical analysis of imaging data

The seed-based functional connectivity analysis was carried out using FILM^55^. For each ROI, a separate single-subject analysis was performed with a single regressor of interest and its temporal derivative to account for non-uniform slice timing and hemodynamic delay, 6 estimated motion parameters and 6 nuisance signal regressors from the white matter and 1 from the ventricles (see Extraction of Time-series section).

In the main group analysis, (1) we analyzed changes in functional connectivity, controlling for each individual’s change in TWSTRS score from W0 to W4 (before and after BoNT-A) by including change in TWSTRS as a covariate; and (2) we also investigated the relationship between the change in functional connectivity with the change in TWSTRS score. To this end, pair-wise within-subject differences were modeled with an additional (explanatory) variable consisting of the corresponding within-subject changes in TWSTRS. The purpose of the (explanatory) variable was twofold: (1) to serve as a regressor (covariate) controlling for the individual variability in clinical effect when assessing the average changes in functional connectivity, and (2) to serve as a predictor of changes specifically linked to the clinical effect of BoNT-A. The second approach exploits the individual variability in treatment response, which is less likely to be affected by other factors that could bias the average differences. To incorporate both pair-wise differences and the TWSTRS change regressor/predictor, a two-step analysis was employed. Within-subject differences were first computed using a fixed effects analysis. The estimated beta values and variances were then carried over to the final mixed effects (fixed effects + random effects) analysis with group mean as the first regressor and the TWSTRS change as the second one. The final step thus yielded 2 pairs of contrasts: W0 > W4 and W4 > W0; Positive Correlation with TWSTRS Change and Negative Correlation with TWSTRS Change. The final mixed effects analysis was performed using FLAME (FMRIB’s Local Analysis of Mixed Effects) with the “stage 1” setting^54^. The whole-brain analysis was limited to the MNI standard brain mask^24^ minus a white-matter mask as described elsewhere^27^. The Z (Gaussianized T statistic images were thresholded using clusters determined by Z > 3 and family-wise error (FWE and Bonferroni corrected (accounting for the number of ROI and two contrasts per contrast pair cluster significance threshold was *p* < 0.00096 (calculated as 0.05 / [26 * 2]. In the exploratory evaluation of the non-hypothesized cortical and subcortical ROIs (supplementary analysis, we applied the same Bonferroni correction as in the main analysis (see Supplementary Methods, similar to the exploratory approach described by Delnooz et al. ^12^.

Significant clusters were anatomically classified according to an overlap with the Harvard–Oxford Cortical and Subcortical Structural Atlases^13^, and the Probabilistic Cerebellar Atlas labels^15^. The resulting statistical images were rendered in Mango v4.0 (Research Imaging Institute, UT Health Science Center at San Antonio, TX, United States).

### Post-hoc plots

To determine the type of change detected using the group contrasts, such as loss of positive or negative functional connectivity, gain of positive or negative connectivity, or both, a *post-hoc* analysis was performed using the masks of significant clusters in group analysis. First, mean Z scores were extracted from individual statistical maps using Featquery tool (part of FSL), which incorporates the back-transformation of the cluster masks from the standard into the individual functional space. Finally, the values were visualized as box plots (contrasts W0 > W4 and W4 > W0) or scatter plots with linear fit (contrasts Positive Correlation with TWSTRS Change and Negative Correlation with TWSTRS Change).

### *Post-hoc* visualization of the average connectivity of cerebellar ROIs

To determine the functional role of the ROIs with significant effects in the main analysis, a *post-hoc* connectivity analysis was performed. The average effects over the two sessions (W0 + W4) were pooled to visualize the functional networks associated with individual ROIs regardless of treatment. No Bonferroni correction was applied to the *post-hoc* statistical maps (mean connectivity) produced solely for visualization purposes. Additionally, to allow interpretation, the cluster-forming threshold was adjusted retrospectively to produce visually comparable co-activation levels across all functional connectivity maps.

## Results

### Clinical data

All patients were injected into the muscles identified by clinical evaluation and by polyelectromyography. The mean total dose of onabotulinumtoxin per patient was 161.8 ± 39.6 IU. The mean value of TWSTRS at Week 0 was 14.3 ± 3.1 and, at Week 4, it was 8.7 ± 3.2 (*p* = 0.00002, one-sided paired t-test), see Table 1. The significant decrease in TWSTRS four weeks after injections suggests a good clinical effect of BoNT treatment.

### Imaging data

**Figure 2:**
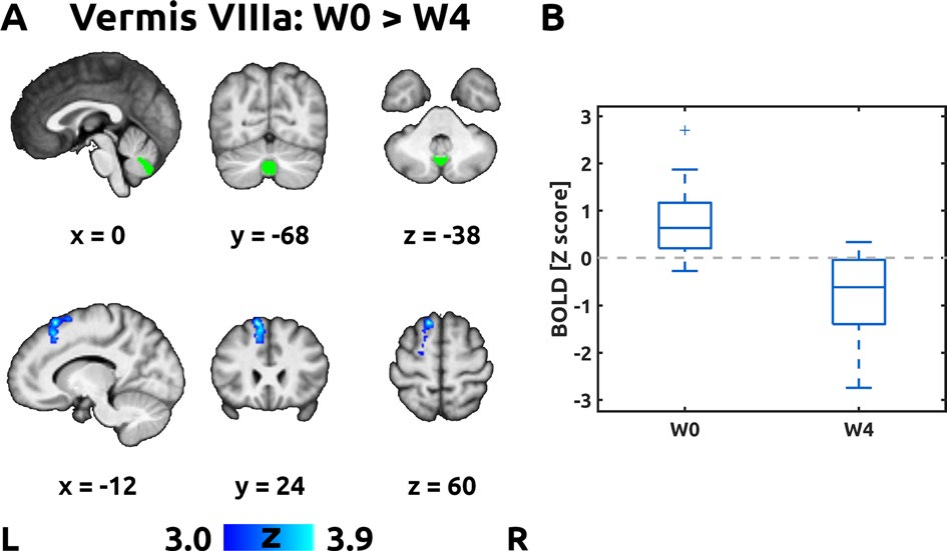
Pair-wise differences in connectivity. Panel A shows significant pair-wise changes in functional connectivity of the seed in the Vermis VIIIa between the baseline visit (W0) and the follow-up (W4), i.e., contrast W4 > W0. No other ROIs showed average changes in functional connectivity after BoNT-A treatment. The green overlay in the top row shows the region of interest in the cerebellum. The blue overlay in the bottom row shows the significant decrease in functional connectivity (positive connectivity turning to negative) in the left superior frontal gyrus, thresholded at Z > 3.0 and Bonferroni-corrected cluster significance level *p* < 0.00096. For remaining conventions, see Fig. 1. Panel B shows box plot of individual blood oxygen level-dependent (BOLD) response (Z score) averaged across the cluster in the left superior frontal gyrus from Panel A.

The first pair of contrasts (W0 > W4 and W4 > W0) revealed a significant decrease in connectivity (W0 > W4) between the vermis lobule VIIIa and the left dorsal mesial frontal cortex (Fig. 2, panel A). The functional connectivity changed from positive to negative after treatment (Fig. 2, panel B). No other ROIs showed average changes in functional connectivity after BoNT-A treatment. This result represents an average effect of the BoNT-A treatment after correcting for inter-individual differences in the clinical effect (determined as a change in TWSTRS). In other words, the functional connectivity decreased after treatment regardless of actual clinical improvement.

**Figure 3:**
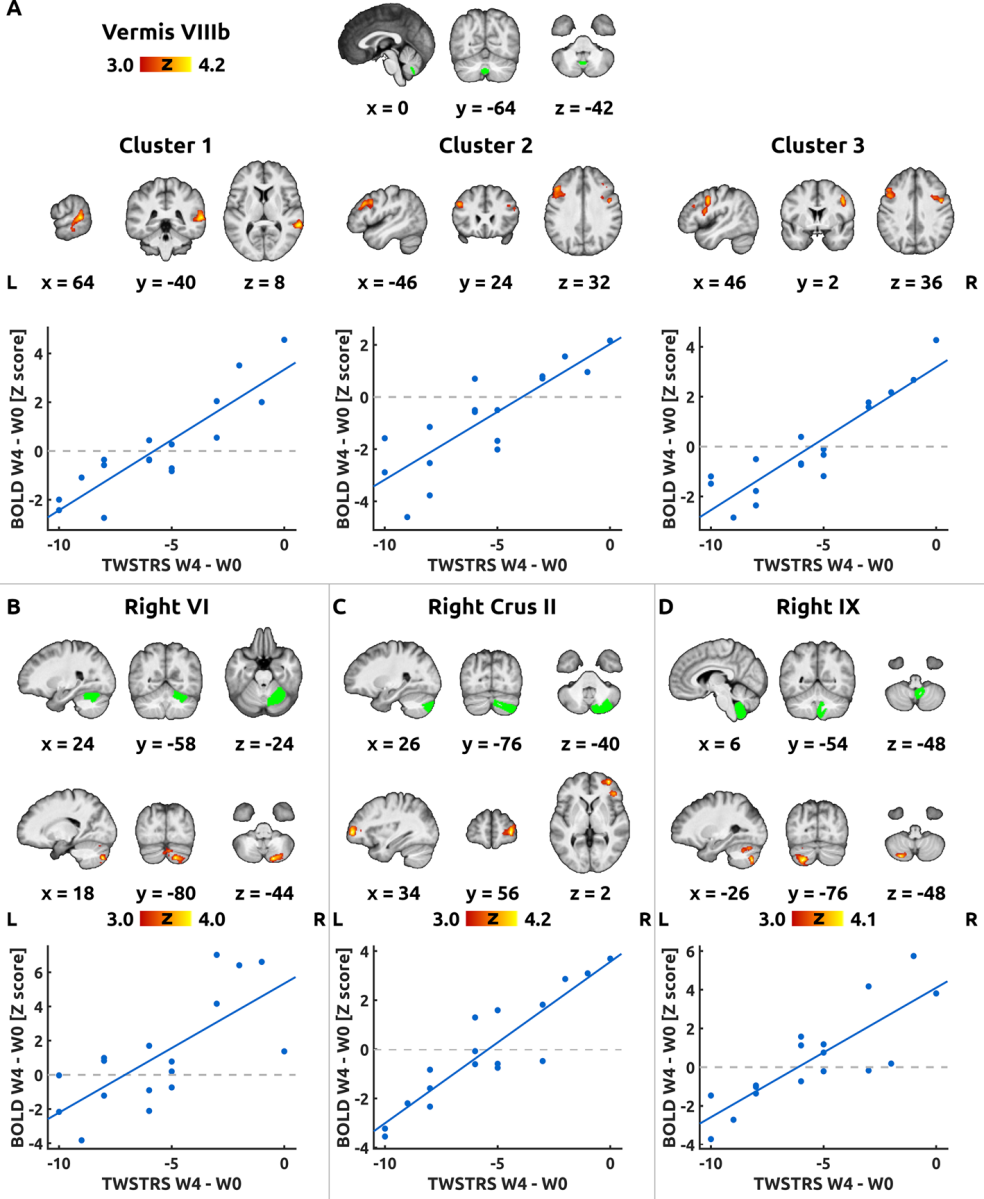
Correlations between the functional connectivity and TWSTRS. Figure shows significant clusters of correlation between the changes in Toronto Western Spasmodic Torticollis Rating Scale (TWSTRS) and changes in functional connectivity from the baseline visit (W0) to the follow-up (W4). Each panel shows significant clusters for a different region of interest (ROI): (**A**) Vermis VIIIb, (**B**) Right Lobule VI, (**C**) Right Crus II, and (**D**) Right Lobule IX. The green overlays in the top row in each panel show the ROI in the cerebellum. The red-yellow overlays in the bottom rows show the significant clusters, thresholded at Z > 3.0 and Bonferroni-corrected cluster significance level p < 0.00096. Below each map, a scatter plot of TWSTRS change in the abscissa against blood oxygen level-dependent (BOLD) response (Z score) in the ordinate demonstrates the individual values. A linear fit line was added for visualization purposes. For remaining conventions, see Fig. 1.

Changes in functional connectivity that were correlated with the individual differences in clinical effect were assessed using the second set of contrasts (Positive Correlation with TWSTRS Change and Negative Correlation with TWSTRS Change). Positive correlations between the connectivity differences (W0 > W4) and the clinical improvement (TWSTRS W0 > W4) were detected for the right lobule VI, right crus II, vermis VIIIb and the right lobule IX (Fig. 3). No significant negative correlation was observed after Bonferroni correction. All clusters of treatment-related differences and correlations (including those not reaching significance after correction) are summarized in Table 2.

**Table 2:** List of significant clusters in the contrasts PreW0 – > W4, W4 > W0, Post contrast and correlation with TWSTRS change.

Seed	Contrast	Cluster Index	Cluster *P *value	Volume (cm^3^)	Z_max_	Z_max_ MNI coordinates	Atlas
Right VI	Positive corr	**1**	**8.30E−04**	4.08	3.99	18–80–44	79.8% R CRBL Crus II
							10.0% R CRBL VIIb
Right Crus II	Positive corr	**1**	**8.24E−05**	6.11	4.20	34 56 2	63.7% R Frontal Pole
							18.6% R Inferior Frontal G, p.t
							12.7% R Inferior Frontal G, p.o
		2	1.34E − -02	2.62	3.68	− 40 38 8	70.7% L Frontal Pole
							17.4% L Middle Frontal G
							6.1% L Inferior Frontal G, p.t
Vermis VIIIa	AW0 > BW4	1	**6.77E−04**	3.21	3.92	− 12 24 60	92.3% L Superior Frontal G
							7.7% L Paracingulate G
Vermis VIIIb	Positive corr	1	**1.54E−04**	4.20	4.16	64−40 8	31.6% R Supramarginal G, p.d
							28.2% R Middle Temporal G, t-o.p
							22.5% R Middle Temporal G, p.d
							14.9% R Superior Temporal G, p.d
		2	**3.19E−04**	3.81	3.84	−46 24 32	86.3% L Middle Frontal G
							8.4% L Inferior Frontal G, p.o
							5.0% L Precentral G
		3	**4.46E−04**	3.63	3.96	46 2 36	44.1% R Precentral G
							30.0% R Middle Frontal G
							25.3% R Inferior Frontal G, p.o
		4	1.39E−03	3.06	4.31	34–76−46	50.5% R CRBL Crus II
							22.5% R CRBL VIIb
							19.4% R CRBL Crus I
							6.5% R CRBL VI
		5	5.39E−03	2.42	3.84	−6 − 74 −28	53.6% L CRBL VI
							30.1% CRBL Vermis VI
							22.5% L Lingual G
							8.6% R Lingual G
							6.0% L Occipital Fusiform G
							5.3% L CRBL Crus I
		6	5.39E−03	1.74	3.56	46 22–6	60.1% R Frontal Pole
							20.6% R Frontal Orbital C
							14.2% R Inferior Frontal G, p.t
		7	3.09E−02	1.66	3.75	−56–48 4	46.2% L Middle Temporal G, t-o.p
							34.1% L Planum Temporale
							16.8% L Superior Temporal G, p.d
Right IX	Positive corr	1	**6.57E−04**	4.16	4.13	−26–76 −48	32.5% L CRBL Crus II 31.5% L CRBL Crus I 20.6% L CRBL VI 15.4% L CRBL VIIb
		2	4.76E−02	1.76	4.06	8−34–42	100% Brain-Stem

Table lists significant t-test clusters in the contrast W0 > W4, W4 > W0, positive, and negative correlation (corr.) with TWSTRS (Toronto Western Spasmodic Torticollis Rating Scale) change. Bold indicates clusters significant after Bonferroni correction. Anatomical labels with the highest probability per voxel are provided including the proportion of labeled voxels. Only labels consisting at least 5% of activated voxels are shown. Note that cerebellar labels may overlap with cortical labels. Abbreviations: C, cortex; CRBL, cerebellum; G, gyrus; L, left; MNI, Montréal Neurological Institute; p.d., posterior division; p.o., pars opercularis; p.t., pars triangularis; R, right; t-o.p., temporooccipital part; W0, Week 0; W4, Week 4; Z_max_, maximum Z score.

There were no significant effects after Bonferroni correction in the supplementary analysis of cortical and subcortical ROIs, see Supplementary Results and Supplementary Table S1.

The *post-hoc* visualization of average connectivity (W0 + W4) for those ROIs with significant clusters in the main analysis is displayed in Fig. 1. Lobule VI was functionally associated predominantly with the bilateral premotor cortices, supplementary motor area (SMA), anterior cingulate cortex, and bilateral intraparietal sulcus; the right crus II, vermis VIIIb and right lobule IX showed mainly connectivity with the bilateral posterior cingulate cortex, precuneus, parietooccipital and mesial prefrontal cortex; vermis VIIIa showed mainly connectivity with the anterior cingulate cortex and the right temporoparietal junction (Fig. 1).

## Discussion

In presented study, we investigated alterations in cerebellar functional connectivity and the possible modifying effect of BoNT injections in CD by means of resting-state fMRI and atlas-based parcellation.

It has been repeatedly suggested that CD is a network disorder^4,35,39,46^. Previous studies reported rather diffuse or non-overlapping structural^25,44,49^ and functional^10,35,47^ changes in multiple brain regions, including the sensorimotor cortices, basal ganglia, thalamus, and, more recently, cerebellum. In fact, it has been suggested that dystonia is caused by a combined dysfunction of several network nodes or their abnormal connectivity^39^. However, it remains unknown, which option describes the best the pathophysiology of dystonia^35^.

The role of the cerebellum in the pathophysiology of CD is still controversial as it is not clear whether functional changes in the cerebellum are the source or a consequence of dystonia^38^. Still, cerebellum showed consistent post-mortem histopathological changes across 6 patients with CD^45^ and was one of the two brain regions specifically associated with secondary CD^9^. Although damage to the cerebellum is usually associated with negative symptoms (a loss of function), it can be argued that different kinds or localizations of lesions may produce different clinical presentations based on the affected pathways^38^. Abnormalities in the anterior lobe of the cerebellum may be associated with dysfunction of the cortical sensorimotor areas in dystonia^43^. Filip et al.^20^ have found cerebellar dysfunction mainly localized in the left posterior hemisphere. They have also demonstrated that CD was associated with decreased functional connectivity between the left cerebellar lobule VI and the contralateral prefrontal cortex, and between the left cerebellar crus I and the ipsilateral middle temporal gyrus. However, the study by Filip et al.^20^ analyzed cerebellar connectivity during a visuospatial task and only from a limited number of seeds (lobule VI, crus I and vermis VIIb), therefore the results are not directly comparable to ours.

In this study, we observed that resting-state connectivity from a number of cerebellar seeds in the posterior vermis and right posterior cerebellar hemisphere decreased with treatment. Moreover, the higher was the clinical benefit of the treatment, the larger was the decrease in functional connectivity (Fig. 3). The cerebellar seeds associated with these changes were found in the following areas: right lobule VI, right crus II, vermis VIIIa and VIIIb, and right lobule IX.

In agreement with previous data in healthy individuals^7^, the mean functional connectivity maps in Fig. 1 demonstrate that lobule VI is connected to the somatomotor, ventral attention, dorsal attention and frontoparietal control networks (Yeo et al., 2011), whereas the crus II and lobule IX predominantly connect to the frontoparietal control (crus II) and default mode network (both crus II and lobule IX) as defined by Yeo et al*.* (2011). Although the lobule VIII has been implicated in motor control^7,16^, in our cohort, vermis VIIIa was associated with the nodes of the ventral attention network, while vermis VIIIb was connected to the hubs of the default mode network (Yeo et al. 2011).

Among the observed treatment-related effects, one stood out: the connectivity from the vermis VIIIa to the left superior frontal gyrus decreased irrespective of the individual differences in the clinical outcome (Fig. 2). Superior frontal gyrus (BA6/9/10) has been reported as one of the regions showing higher resting-state functional connectivity in CD than in the control group, for a compound cortico-subcortical motor seed^12,35^ or sensorimotor cortex^19^, and has also manifested different structural connectivity pattern^26^ and increased gray matter volume^37^ in other focal dystonias. Our treatment-related effect might be relevant, though possibly related to secondary phenomena following the BoNT treatment, e.g., behavioral or affective changes associated with motor improvement.

However, the remaining significant changes in connectivity scaled with the degree of clinical motor improvement. The change in TWSTRS was either correlated with the strength of intra-cerebellar functional connections, as in the case of seeds in the right lobule VI and right lobule IX, or with cerebro-cerebellar connections, as in the case of seeds in the right crus II and vermis VIIIb (Fig. 3).

The right lobule VI, which is predominantly associated with the sensorimotor control areas, showed in good responders decreasing connectivity with the right crus II (Fig. 3, panel B), a cerebellar region associated with the default mode network^7^. In contrast, the right lobule IX showed decreasing connectivity with the left crus II (Fig. 3, panel D). In this case, both regions are functionally associated with the default mode network^7^.

With respect to the cerebro-cerebellar connections, the clinical improvement (change in TWSTRS) was correlated with the functional connectivity strength between the right crus II and the right lateral prefrontal cortex, i.e., a component of the frontoparietal control network^53^. Furthermore, correlation with change in TWSTRS was observed between the vermis VIIIb and clusters in the right temporoparietal junction, left middle frontal gyrus, and right premotor cortex, which represent nodes of the ventral attention, frontoparietal control, and dorsal attention networks, respectively^53^, Yeo et al. (2011).

In summary, BoNT injection followed by successful control of CD signs led to (1) decrease in intrinsic connectivity in the posterior cerebellum, including connectivity between motor and more cognitive regions of the cerebellum. Furthermore, (2) the vermis VIIIa and VIIIb, implicated in motor control, showed decreased connectivity with bilateral dorsolateral frontal and premotor and right temporoparietal cortices, and (3) the crus II, predominantly involved in cognitive functions, showed decreased connectivity with the right prefrontal cortex.

The decreasing cerebro-cerebellar connectivity after treatment is in line with evidence for abnormally “increased” (i.e., less negative) connectivity in CD between the sensorimotor cortex and a cerebellar seed in vermis defined as an overlap of dystonia-related brain lesions^9^. Our findings also have to be considered in the context of previous published papers on central treatment effects in idiopathic dystonia e.g.,^12,18,38^.

Our previous study showed decreased activation during complex finger tapping task throughout the sensorimotor system after BoNT treatment, including the anterior cerebellum^38^. Although this could be viewed as decreasing task-driven cerebro-cerebellar connectivity, thus compatible with our current results, a direct comparison of task-related and resting-state data is not straightforward. A previous resting-state fMRI study in CD patients that assessed BoNT effect revealed no difference in the cerebellar network^12^. However, cerebellar connectivity was assessed in that study on a global scale, de facto using a single cerebellar seed. Though reasonable in search for large effects, such approach could possibly miss changes on a fine scale of individual cerebellar lobuli.

Further evidence for the role of the cerebellum comes from structural imaging studies. In patients with various types of dystonia chronically treated with deep brain stimulation (DBS), voxel-based morphometry revealed increased gray matter density in the cerebellar vermis, supplementary motor area (SMA) and anterior cingulate cortex, which was more profound in good responders^18^. Together with our data, this study suggests a prominent role of the cerebellum and its frontal connections in the normalization of the motor function, though the exact mechanisms in DBS and BoNT may considerably differ.

It should be acknowledged that the mechanism of BoNT effect on central structures has not been fully elucidated. The established mechanisms are summarized by Marchand-Pauvert et al.^36^, including blockade of the gamma motor endings, plasticity evoked by blockade of the neuromuscular transmission, and retrograde transport and transcytosis of BoNT. Although it is not entirely clear, which of the mechanisms plays a key role in the clinical improvement after BoNT, there is multiple evidence from recent studies about modulation of activity of various structures in the central nervous system after BoNT both in motor and sensory areas. We suggest that in focal dystonias, BoNT-induced effects encompass complex mechanisms beyond chemodenervation of the injected muscles^5^.

In conclusion, the presented data provide evidence for modulation of cortico-cerebellar connectivity resulting from successful treatment by BoNT.

## Supplementary Information

Supplementary Information

## Abbreviations

BoNT: Botulinum neurotoxin A
CD: Cervical dystonia
DBS: Deep brain stimulation
EPI: Echo-planar imaging
FLAME: FMRIB’s Local Analysis of Mixed Effects
fMRI: Functional MRI
FoV: Field of view
FSL: FMRIB’s Software Library
FEW: Family-wise error
MR: Magnetic resonance
ROI: Regions of interest
SMA: Supplementary motor area
TR/TE: Repetition time/echo time
TWSTRS: Toronto Western Spasmodic Torticollis Rating Scale
W0: Week 0
W4: Week 4

## References

[1] Abbruzzese G, Berardelli A (2006). Neurophysiological effects of botulinum toxin type A. Neurotox Res..

[2] Avanzino L, Abbruzzese G (2012). How does the cerebellum contribute to the pathophysiology of dystonia?. Basal Ganglia Knowl. Gaps Parkinson’s Dis. Other Movement Disorders.

[3] Balint B, Mencacci NE, Valente EM, Pisani A, Rothwell J, Jankovic J, Vidailhet M, Bhatia KP (2018). Dystonia. Nat. Rev. Dis. Primers.

[4] Battistella G, Termsarasab P, Ramdhani RA, Fuertinger S, Simonyan K (2017). Isolated focal dystonia as a disorder of large-scale functional networks Cereb. Cortex.

[5] Berardelli A, Conte A (2019). Dystonias. Handb. Exp. Pharmacol..

[6] Brodoehl S, Wagner F, Prell T, Klingner C, Witte OW, Günther A (2019). Cause or effect: Altered brain and network activity in cervical dystonia is partially normalized by botulinum toxin treatment. NeuroImage Clin..

[7] Buckner RL, Krienen FM, Castellanos A, Diaz JC, Yeo BTT (2011). The organization of the human cerebellum estimated by intrinsic functional connectivity. J. Neurophysiol..

[8] Consky E, Lang A, Jankovic J (1994). Clinical Assessments of patients with Cervical Dystonia. Therapy with Botulinum Toxin.

[9] Corp DT, Joutsa J, Darby RR, Delnooz CCS, van de Warrenburg BPC, Cooke D, Prudente CN, Ren J, Reich MM, Batla A, Bhatia KP, Jinnah HA, Liu H, Fox MD (2019). Network localization of cervical dystonia based on causal brain lesions. Brain.

[10] de Vries PM, Johnson KA, de Jong BM, Gieteling EW, Bohning DE, George MS, Leenders KL (2008). Changed patterns of cerebral activation related to clinically normal hand movement in cervical dystonia. Clin. Neurol. Neurosurg..

[11] Delnooz CCS, Pasman JW, Beckmann CF, van de Warrenburg BPC (2015). Altered striatal and pallidal connectivity in cervical dystonia. Brain Struct. Funct..

[12] Delnooz CCS, Pasman JW, Beckmann CF, van de Warrenburg BPC (2013). Task-free functional MRI in cervical dystonia reveals multi-network changes that partially normalize with botulinum toxin. PLoS ONE.

[13] Desikan RS, Ségonne F, Fischl B, Quinn BT, Dickerson BC, Blacker D, Buckner RL, Dale AM, Maguire RP, Hyman BT, Albert MS, Killiany RJ (2006). An automated labeling system for subdividing the human cerebral cortex on MRI scans into gyral based regions of interest. Neuroimage.

[14] Deuschl G, Heinen F, Kleedorfer B, Wagner M, Lücking CH, Poewe W (1992). Clinical and polymyographic investigation of spasmodic torticollis. J. Neurol..

[15] Diedrichsen J, Balsters JH, Flavell J, Cussans E, Ramnani N (2009). A probabilistic MR atlas of the human cerebellum. Neuroimage.

[16] Diedrichsen J, King M, Hernandez-Castillo C, Sereno M, Ivry RB (2019). Universal transform or multiple functionality? Understanding the contribution of the human cerebellum across task domains. Neuron.

[17] Eickhoff SB, Paus T, Caspers S, Grosbras M-H, Evans AC, Zilles K, Amunts K (2007). Assignment of functional activations to probabilistic cytoarchitectonic areas revisited. Neuroimage.

[18] Fečíková A, Jech R, Čejka V, Čapek V, Šťastná D, Štětkářová I, Mueller K, Schroeter ML, Růžička F, Urgošík D (2018). Benefits of pallidal stimulation in dystonia are linked to cerebellar volume and cortical inhibition. Sci. Rep..

[19] Feng L, Yin D, Wang X, Xu Y, Xiang Y, Teng F, Pan Y, Zhang X, Su J, Wang Z, Jin L (2020). Brain connectivity abnormalities and treatment-induced restorations in patients with cervical dystonia. Eu. J. Neurol..

[20] Filip P, Gallea C, Lehéricy S, Bertasi E, Popa T, Mareček R, Lungu OV, Kašpárek T, Vaníček J, Bareš M (2017). Disruption in cerebellar and basal ganglia networks during a visuospatial task in cervical dystonia.

[21] Filip P, Lungu OV, Bareš M (2013). Dystonia and the cerebellum: a new field of interest in movement disorders?. Clin. Neurophysiol..

[22] Gelb DJ, Yoshimura DM, Olney RK, Lowenstein DH, Aminoff MJ (1991). Change in pattern of muscle activity following botulinum toxin injections for torticollis. Ann. Neurol..

[23] Gilio F, Currà A, Lorenzano C, Modugno N, Manfredi M, Berardelli A (2000). Effects of botulinum toxin type A on intracortical inhibition in patients with dystonia. Ann. Neurol..

[24] Grabner G, Janke AL, Budge MM, Smith D, Pruessner J, Collins DL (2006). Symmetric atlasing and model based segmentation: an application to the hippocampus in older adults. Med. Image Comput. Comput. Assist. Interv..

[25] Gracien R-M, Petrov F, Hok P, van Wijnen A, Maiworm M, Seiler A, Deichmann R, Baudrexel S (2019). Multimodal quantitative MRI reveals no evidence for tissue pathology in idiopathic cervical dystonia. Front. Neurol..

[26] Hanekamp S, Simonyan K (2020). The large-scale structural connectome of task-specific focal dystonia. Hum. Brain Mapp..

[27] Hok P, Opavský J, Labounek R, Kutín M, Šlachtová M, Tüdös Z, Kaňovský P, Hluštík P (2019). Differential effects of sustained manual pressure stimulation according to site of action. Front. Neurosci..

[28] Jankovic J (2004). Treatment of cervical dystonia with botulinum toxin. Mov. Disord..

[29] Jenkinson M, Beckmann CF, Behrens TEJ, Woolrich MW, Smith SM (2012). FSL. Neuroimage.

[30] Jiang W, Lei Y, Wei J, Yang L, Wei S, Yin Q, Luo S, Guo W (2019). Alterations of interhemispheric functional connectivity and degree centrality in cervical dystonia: a resting-state fMRI study. Neural Plast..

[31] Jost WH, Schramm A, Müngersdorf M, Stenner A, Schwingenschuh P, Maisonobe P (2019). Effectiveness of botulinum neurotoxin type A injections in naïve and previously-treated patients suffering from Torti- or Laterocollis or -caput: Results from a German-Austrian open-label prospective post-marketing surveillance study. J. Neurol. Sci..

[32] Kaňovský P, Dufek J, Halačková H, Rektor I (1997). Change in the pattern of cervical dystonia might be the cause of benefit loss during botulinum toxin treatment. Eur. J. Neurol..

[33] Kaňovský P, Streitová H, Dufek J, Znojil V, Daniel P, Rektor I (1998). Change in lateralization of the P22/N30 cortical component of median nerve somatosensory evoked potentials in patients with cervical dystonia after successful treatment with botulinum toxin A. Mov. Disord..

[34] Lehéricy S, Tijssen MAJ, Vidailhet M, Kaji R, Meunier S (2013). The anatomical basis of dystonia: current view using neuroimaging. Mov. Disord..

[35] Li Z, Prudente CN, Stilla R, Sathian K, Jinnah HA, Hu X (2017). Alterations of resting-state fMRI measurements in individuals with cervical dystonia. Hum. Brain Mapp..

[36] Marchand-Pauvert V, Aymard C, Giboin L-S, Dominici F, Rossi A, Mazzocchio R (2013). Beyond muscular effects: depression of spinal recurrent inhibition after botulinum neurotoxin A. J. Physiol. (Lond.).

[37] Martino D, Di Giorgio A, D'Ambrosio E, Popolizio T, Macerollo A, Livrea P, Bertolino A, Defazio G (2011). Cortical gray matter changes in primary blepharospasm: a voxel-based morphometry study. Mov. Disord..

[38] Nevrlý M, Hluštík P, Hok P, Otruba P, Tüdös Z, Kaňovský P (2018). Changes in sensorimotor network activation after botulinum toxin type A injections in patients with cervical dystonia: a functional MRI study. Exp. Brain Res..

[39] Neychev VK, Gross RE, Lehéricy S, Hess EJ, Jinnah HA (2011). The functional neuroanatomy of dystonia. Neurobiol. Dis..

[40] Norris SA, Morris AE, Campbell MC, Karimi M, Adeyemo B, Paniello RC, Snyder AZ, Petersen SE, Mink JW, Perlmutter JS (2020). Regional, not global, functional connectivity contributes to isolated focal dystonia. Neurology.

[41] Opavský R, Hluštík P, Otruba P, Kaňovský P (2012). Somatosensory cortical activation in cervical dystonia and its modulation with botulinum toxin: an fMRI study. Int. J. Neurosci..

[42] Opavský R, Hluštík P, Otruba P, Kaňovský P (2011). Sensorimotor network in cervical dystonia and the effect of botulinum toxin treatment: A functional MRI study. J. Neurol. Sci. Spec. Section ECF 2009 A New Treatment Era Muliple Sclerosis Options Challenges Risks Europ Charcot Foundation Symp..

[43] Piccinin CC, Santos MCA, Piovesana LG, Campos LS, Guimarães RP, Campos BM, Torres FR, França MC, Amato-Filho AC, Lopes-Cendes I, Cendes F, D’Abreu A (2014). Infratentorial gray matter atrophy and excess in primary craniocervical dystonia. Parkinsonism Relat. Disord..

[44] Prell T, Peschel T, Koehler B, Bokemeyer MH, Dengler R, Guenther A, Grosskreutz J (2013). Structural brain abnormalities in cervical dystonia. BMC Neurosci..

[45] Prudente CN, Pardo CA, Xiao J, Hanfelt J, Hess EJ, LeDoux MS, Jinnah HA (2013). Neuropathology of cervical dystonia. Exp. Neurol..

[46] Prudente CN, Hess EJ, Jinnah HA (2014). Dystonia as a network disorder: what is the role of the cerebellum?. Neuroscience.

[47] Prudente CN, Stilla R, Singh S, Buetefisch C, Evatt M, Factor SA, Freeman A, Hu XP, Hess EJ, Sathian K, Jinnah HA (2016). A functional magnetic resonance imaging study of head movements in cervical dystonia. Front. Neurol..

[48] Pruim RHR, Mennes M, van Rooij D, Llera A, Buitelaar JK, Beckmann CF (2015). ICA-AROMA: A robust ICA-based strategy for removing motion artifacts from fMRI data. Neuroimage.

[49] Ramdhani RA, Kumar V, Velickovic M, Frucht SJ, Tagliati M, Simonyan K (2014). What’s special about task in dystonia? A voxel-based morphometry and diffusion weighted imaging study. Mov. Disord..

[50] Sadnicka A, Hoffland BS, Bhatia KP, van de Warrenburg BP, Edwards MJ (2012). The cerebellum in dystonia - help or hindrance?. Clin. Neurophysiol..

[51] Sarasso E, Agosta F, Piramide N, Bianchi F, Butera C, Gatti R, Amadio S, Del Carro U, Filippi M (2020). Sensory trick phenomenon in cervical dystonia: a functional MRI study. J. Neurol..

[52] Shakkottai VG, Batla A, Bhatia K, Dauer WT, Dresel C, Niethammer M, Eidelberg D, Raike RS, Smith Y, Jinnah HA, Hess EJ, Meunier S, Hallett M, Fremont R, Khodakhah K, LeDoux MS, Popa T, Gallea C, Lehericy S, Bostan AC, Strick PL (2017). Current opinions and areas of consensus on the role of the cerebellum in dystonia. Cerebellum.

[53] Vincent JL, Kahn I, Snyder AZ, Raichle ME, Buckner RL (2008). Evidence for a frontoparietal control system revealed by intrinsic functional connectivity. J. Neurophysiol..

[54] Woolrich MW, Behrens TEJ, Beckmann CF, Jenkinson M, Smith SM (2004). Multilevel linear modelling for FMRI group analysis using Bayesian inference. Neuroimage.

[55] Woolrich MW, Ripley BD, Brady M, Smith SM (2001). Temporal autocorrelation in univariate linear modeling of FMRI data. Neuroimage.

