# Supplementary material for: Botulinum toxin injection changes resting state cerebellar connectivity in cervical dystonia
Source: Sci Rep. 2021 Apr 15;11:8322. doi: 10.1038/s41598-021-87088-z (PMC8050264; doi:10.1038/s41598-021-87088-z)
Supplement: Supplementary file 1 — Supplementary Information [file 41598_2021_87088_MOESM1_ESM.docx]

**Botulinum toxin injection changes resting state cerebellar connectivity in cervical dystonia.**

Supplementary Information

**Authors:**

Pavel Hok^1,*^, Lenka Hvizdošová^1,*^, Pavel Otruba^1^, Michaela Kaiserová^1^, Markéta Trnečková^1,3^, Zbyněk Tüdös^2^, Petr Hluštík^1^, Petr Kaňovský^1^, Martin Nevrlý^1,†^

**Institutions:**

1 Department of Neurology, University Hospital and Faculty of Medicine and Dentistry of Palacký University Olomouc, Olomouc, Czech Republic

2 Department of Radiology, University Hospital and Faculty of Medicine and Dentistry of Palacký University Olomouc, Olomouc, Czech Republic

3 Department of Computer Science, Faculty of Science of Palacký University Olomouc, Olomouc, Czech Republic

* Both authors contributed equally to this work

† Corresponding author

**Supplementary Methods**

To provide a broader context for the changes in the cerebellar networks, we performed a supplementary connectivity analysis using several regions of interest in the cerebral cortex and basal ganglia selected *a priori* as a reference. The specific procedures of the supplementary analysis are described below, the remaining procedures were identical to the main analysis and are described in the main text.

**Extraction of Time-series:**

Cerebral regions associated with focal dystonia other than the cerebellum were chosen based on the available literature: primary motor cortex, primary somatosensory cortex, and basal ganglia (Corp et al., 2019; Jinnah and Hess, 2006; Neychev et al., 2008). The cortical regions were extracted from the Jülich histological atlas (Geyer et al., 2000, 1996; Grefkes et al., 2001), and a probabilistic atlas of the basal ganglia (Keuken and Forstmann, 2015) using a 25% probability threshold. The cortical regions of interest (ROIs) consisted of the following atlas labels (left and right regions were included as separate ROIs): primary motor cortex Brodmann area (BA) 4a, primary motor cortex BA4p, primary somatosensory cortex BA1, primary somatosensory cortex BA2, primary somatosensory cortex BA3a, and primary somatosensory cortex BA3b. The basal ganglia ROIs consisted of the following atlas labels (left and right regions were included as separate ROIs): red nucleus, substantia nigra, subthalamic nucleus, striatum, globus pallidus externa, and globus pallidus interna. After resampling into the individual space, the masks of the subthalamic nuclei have been discarded as they contained 0 voxels in multiple subjects. The supplementary set of ROIs thus consisted of 22 regions in total. Next, a whole-brain seed-based functional connectivity analysis was performed for each of the ROIs, whjle all remaining procedures were the same as for the main analysis.

**Statistical Analysis of Imaging Data:**

The Z (Gaussianized T) statistic images were thresholded using clusters determined by Z > 3 and family-wise error (FWE) and Bonferroni corrected (accounting for the number of ROI and two contrasts per contrast pair) cluster significance threshold was p < 0.0011 (calculated as 0.05 / [22 * 2]). All other procedures were the same for both main and supplementary set of ROIs.

**Supplementary Results**

After Bonferroni correction, there was neither any significant change in contrasts W0 > W4 and W4 > W0, nor any significant correlation (contrasts Positive Correlation with TWSTRS Change and Negative Correlation with TWSTRS Change, see main body test for the definition of contrasts). All clusters of treatment-related differences and correlations (including those not reaching significance after correction) are summarized in Supplementary Table S1.

**Supplementary Table S1**. List of significant clusters in the contrasts W0 > W4, W4 > W0, and correlation with TWSTRS change for cortical and subcortical regions of interest.

| **Seed** | **Contrast** | **Cluster Index** | **Cluster P value** | **Volume [cm^3^]** | **Z_max_** | **Z_max_ MNI coordinates** | **Atlas** |
| --- | --- | --- | --- | --- | --- | --- | --- |
| Left SN | negative corr. | 1 | 4.39E-02 | 1.40 | 3.66 | 28 −26 52 | 52.0% R Postcentral G 44.0% R Precentral G |
| Right M1 (BA4p) | negative corr. | 1 | 3.33E-02 | 1.78 | 4.53 | −46 −70 −30 | 94.6% L CRBL Crus I 22.4% L Occipital Fusiform G 11.2% L Lateral Occipital C, i.d. |
| Table lists significant t-test clusters of the supplementary analysis in the contrast W0 > W4, W4 > W0, positive, and negative correlation (corr.) with TWSTRS (Toronto Western Spasmodic Torticollis Rating Scale) change. There were no significant clusters after Bonferroni correction (p < 0.0011). Anatomical labels with the highest probability per voxel are provided including the proportion of labeled voxels. Only labels consisting at least 5% of activated voxels are shown. Note that cerebellar labels may overlap with cortical labels. Abbreviations: BA4p, Brodmann area 4p; C, cortex; CRBL, cerebellum; G, gyrus; i.d., inferior division; L, left; M1, primary motor cortex; MNI, Montréal Neurological Institute; R, right; SN, *substantia nigra*; W0, Week 0; W4, Week 4; Z_max_, maximum Z score. | | | | | | | |

**References**

Corp, D.T., Joutsa, J., Darby, R.R., Delnooz, C.C.S., van de Warrenburg, B.P.C., Cooke, D., Prudente, C.N., Ren, J., Reich, M.M., Batla, A., Bhatia, K.P., Jinnah, H.A., Liu, H., Fox, M.D., 2019. Network localization of cervical dystonia based on causal brain lesions. Brain 142, 1660–1674. https://doi.org/10.1093/brain/awz112

Geyer, S., Ledberg, A., Schleicher, A., Kinomura, S., Schormann, T., Bürgel, U., Klingberg, T., Larsson, J., Zilles, K., Roland, P.E., 1996. Two different areas within the primary motor cortex of man. Nature 382, 805–807. https://doi.org/10.1038/382805a0

Geyer, S., Schormann, T., Mohlberg, H., Zilles, K., 2000. Areas 3a, 3b, and 1 of human primary somatosensory cortex. Part 2. Spatial normalization to standard anatomical space. Neuroimage 11, 684–696. https://doi.org/10.1006/nimg.2000.0548

Grefkes, C., Geyer, S., Schormann, T., Roland, P., Zilles, K., 2001. Human somatosensory area 2: observer-independent cytoarchitectonic mapping, interindividual variability, and population map. Neuroimage 14, 617–631. https://doi.org/10.1006/nimg.2001.0858

Jinnah, H.A., Hess, E.J., 2006. A new twist on the anatomy of dystonia: the basal ganglia and the cerebellum? Neurology 67, 1740–1741. https://doi.org/10.1212/01.wnl.0000246112.19504.61

Keuken, M.C., Forstmann, B.U., 2015. A probabilistic atlas of the basal ganglia using 7 T MRI. Data Brief 4, 577–582. https://doi.org/10.1016/j.dib.2015.07.028

Neychev, V.K., Fan, X., Mitev, V.I., Hess, E.J., Jinnah, H.A., 2008. The basal ganglia and cerebellum interact in the expression of dystonic movement. Brain 131, 2499–2509. https://doi.org/10.1093/brain/awn168
